# Organized interests in post-communist policy-making: a new dataset for comparative research

**DOI:** 10.1057/s41309-022-00172-1

**Published:** 2022-11-15

**Authors:** Michael Dobbins, Rafael Pablo Labanino, Rafał Riedel, Szczepan Czarnecki, Brigitte Horváth, Emilia Szyszkowska

**Affiliations:** 1 Department of Politics and Management, University of Konstanz, Universitätsstrasse 10, 78457 Constance, Germany; 2 Katedra Systemów Politycznych i Administracyjnych/Chair for Political and Administrative Systems, Opole University, Collegium Civitas Pokój 312, Opole, Poland

**Keywords:** Organized interests, Central and Eastern Europe, Population ecology, Survey data

## Abstract

This article familiarizes readers with the international research project ‘The Missing Link: Exploring Organized Interests in Post-Communist Policy-Making’ (OrgIntCEE). The project team has focused on how populations of organized interests in the region have evolved, how they interact with state institutions as well as the group-specific characteristics driving access to policy-makers. The project also explores how Europeanization has affected post-communist interest groups as well as other factors contributing to their “coming-of-age.” We provide a comprehensive overview of the population ecology and survey datasets, while shedding light on the challenges during the data collection process. After a short overview of the project context and structure, we present some country-specific aggregated data on organizational densities and their political activity. We also reflect on potential uses for the data, before wrapping up the article with a self-critical assessment of what could have been done differently as a roadmap for future research.

**Supplementary Information:**

The online version contains supplementary material available at 10.1057/s41309-022-00172-1.

## Introduction

This article familiarizes readers with the international research project ‘The Missing Link: Exploring Organized Interests in Post-Communist Policy-Making’ (OrgIntCEE), which has been carried out at the University of Konstanz (Germany) and Opole University (Poland) since 2018.^1^ The OrgIntCEE project can be traced back to an observation by the principal investigators some five years ago that systematic analyses of organized interests in the region, their involvement in policy-making and potential impact on concrete policies have been relatively seldom (for exceptions, see Cox and Vass 2000; Gallai et al. 2015; Fink-Hafner 2011; Novak and Fink-Hafner 2019; Rozbicka et al. 2021a). Great scholarly effort was dedicated to understanding how Central and Eastern European (CEE) countries simultaneously transformed from one-party monopolies to democracy, from communism to capitalism and, in many cases, also created new nation states (Bruszt and Stark 1991; Ramet 2010; Rose 2009). There has also been a wealth of studies on the development of formal electoral institutions (Beliaev 2006; Dimitrov et al. 2006; Easter 1997), post-communist party systems (Kitschelt et al. 1999; Enyedi and Bértoa 2018) and Europeanization processes (Zubek 2011; Agh 1999).

However, it struck us that the previous literature had only insufficiently focused on organized interests. By channeling citizens’ preferences and expertise into policy-making, organized interests are a precondition for any functional democracy. Dysfunctional interest group systems may undermine the democratic process, in particular when rent-seeking special interests monopolize the interest intermediation process to the detriment of other broader civic interests (Olson 1965; Dür and De Bièvre 2007). In other words, transparent and accountable lobbying systems are essential for former Eastern Bloc countries, where democratic consolidation is still a work-in-progress.

The relative lack of research on CEE interest groups is somewhat astounding due to the unique historical trajectory. Communist regimes essentially engineered their own ideology-based civic movements and regulated all forms of civil participation, while marginalizing or outlawing dissident movements and those unaffiliated with the party. Whether rooted within labor unionism, religion or academics, anti-communist activist movements were forced to operate underground, even during more liberal phases of communism (Makowski 2015; Gliński 2006). Nevertheless, as Ekiert and Foa (2011) emphasize, the ‘official’ organizational ecosystem was not merely a means of forced mobilization but served as a ‘transmission belt’ between society and the communist party state.

Amid the geopolitical decline of the Soviet Union, civil society decisively contributed to the collapse of communism. The anti-communist opposition in Poland was fueled by the *Solidarność* workers’ movement, which later also figured prominently in the democratization process. In Czechoslovakia academics, students, artists, and ordinary citizens also allied with trade unions to topple communism. In Hungary, newly formed independent trade unions and other societal groups participated together with anti-communist political parties in the roundtable discussions with their communist counterparts and the Hungarian Socialist Workers’ Party in 1989 resulting in the largely negotiated dismantling of the communist party state. In Slovenia, which already exhibited a relatively sizable and heterogeneous population of interest groups under Yugoslav socialism, various nationalist-separatist movements opposing one-party rule helped steering the nation toward independence and democratization. Throughout the region, civil society mobilization ultimately brought communism to its knees and the early democratization process subsequently heralded an unprecedented boom in organizational formations (Labanino et al. 2021a; Rozbicka et al. 2021a).

Yet during the 1990s, the socioeconomic transition process itself was ultimately often implemented hierarchically without genuine civil society input. Civil society organizations again were often side-lined, while technocratic executives navigated these fragile democracies through the complex challenges of post-communism and Europeanization (Dimitrov et al. 2006). Furthermore, particularly trade unions were haunted by their role in communist party states, and the dismantling of large communist firms and the subsequent privatization process diminished their societal base (Szikra and Tomka 2009; Crowley and Ost 2001; Avdagic 2005). Hence, scholars have argued that the prevalence of strong executives (Zubek 2005) along with clientelist networks (Pleines 2004) and the communist legacy of civic oppression (Cox 2012; Kostelka 2014; Pop-Eleches and Tucker 2011) still impede participative interest representation. Indeed, low associational membership, trust and weak consultative procedures were defining features of the early post-communist decades (Borragán 2006; Ost 2011).

Against this background, CEE democracies faced unparalleled challenges in establishing effective institutions for civic representation (Gliński 2006; Makowski 2015). Already by the mid- to late-1990s though, reform-oriented governments aimed to promote social dialog, appear more responsive to citizens and manage social divisions (Iankova 2002). Multiple new platforms for civil society engagement emerged (Ost 2000), a trend reinforced by the desire to conform to Western European norms. The subsequent European integration process not only brought about opportunities for EU-level mobilization (e.g., Dür and Mateo 2014), but also fostered the spread of European models and learning effects (Grabbe 2001), both regarding individual policies as well as for interest groups in terms of organizational development (Maloney et al. 2018). Therefore, the strength of post-communist organized interests may be underestimated.

However, the region recently also witnessed various crackdowns on civic life amid “democratic backsliding.” Most notably Hungary and Poland, but arguably also Czechia and later Slovenia have retreated from the “avant-garde” of post-communist democratization and embraced increasingly authoritarian governance styles (Bozóki and Hegedűs 2018; Hanley and Vachudova 2018; Przybylski 2018). The 2021 Nations in Transit Report by Freedom House reveals that Hungary and Poland underwent the steepest decline in democratic quality ever recorded (Csaky 2021). Hungary was downgraded first from a stable to semi-consolidated democracy and in 2020 to a ‘transitional hybrid regime.’ Poland is still rated by Freedom House as a semi-consolidated democracy. Although still considered a consolidated democracy, the quality of Czech democracy declined somewhat under the Babiš government (Buštíková 2021). Democratic quality also deteriorated in Slovenia under the third premiership of Janez Janša between 2020 and 2022 (Lovec 2021).

Amid backsliding and ‘cesarean politics’ (Sata and Karolewski 2020), CEE governments have increasingly developed systems of patronism and clientelism characterized by political closure, the predominance of informal networks and elaborate systems of rewards and punishments for civic organizations (Olejnik 2020). Illiberal incumbents may restrict their funding and obstruct their agendas (Carothers 2016; Buyse 2018; Bromley et al. 2020), while weakening or abolishing formal interest intermediation mechanisms (Olejnik 2020). Yet other authors have argued that neo-authoritarian CEE governments are consolidating new “illiberal” civil societies by deliberately nurturing their own organizations (Greskovits 2020; Ekiert 2019). Targeted support for national-conservative, identity-oriented civic organizations is aimed at fending off undesired western support for “liberal” causes. Unequal treatment of various interest groups can undermine core democratic principles and may be interpreted as a symptom, but also as a cause of democracy erosion observed across the region.

Against this background, there are strong reasons for scholars to immerse themselves into the diverse, unpredictable world of organized interests in CEE. This article paves the way for doing so. We continue with a short overview of the project structure, before discussing the compilation of our population ecology datasets. In Sect. 3, we justify our logic for a comparative online survey, present some data on response rates and outline its major building blocks and aggregate country-specific findings. We wrap up the article with a self-critical assessment of what could have been done differently as a roadmap for future research.

## The overarching structure of the OrgIntCEE project

No research project can cover the entire breadth and heterogeneity of interest groups politics, let alone in numerous countries. After all, thousands of organizations of different sizes operate in countless areas of public policy in the enormously vast and heterogeneous post-communist sphere spanning from Berlin to the Far East. Therefore, we narrowed the scope to four countries, three policy areas and five overarching thematic blocks to ensure a certain degree of coherence, which still enabled us to grasp the diversity of interest groups in CEE.

Our case selection comprised Czechia, Hungary, Poland and Slovenia. As new democracies, market economies, and EU members they share similarities, but differ substantially regarding several decisive characteristics for interest group politics: election funding, lobbying regulations and economic coordination. Czechia exhibits a very weakly regulated market economy with privately funded elections and lax lobbying regulations (Šimral 2015; McGrath 2008). The Polish economy is also relatively weakly coordinated. However, elections are publicly funded, and extensive lobbying regulations exist, which may stymie the influence of interest groups (McGrath 2008). Hungary exhibits stronger market coordination (Tarlea 2017; Duman and Kureková 2012), elections are publicly funded, lobbying activities are, however, only loosely regulated since 2010 (European Commission 2020; Laboutková et al. 2020). Slovenia arguably is the most coordinated market economy in CEE (Bohle and Greskovits 2012), but regulatory controls over lobbying, party funding and electoral campaigns are comparatively weak, hence making it a polar opposite case to Poland.

Looking at these countries, we explore three critical policy areas for the viability of post-communist democracies—energy, higher education and health care—each of which is of long-term strategic importance for national security and well-being. Importantly, the policy areas are diverse and enable *within-country* comparative perspectives on interest groups as well as *between-country* comparisons of individual policy areas.

Although the transformation and European integration processes along with the bankruptcy of many energy-intensive industries led to a reduction in energy consumption, most of CEE is still heavily dependent on domestic and imported (Russian) coal and gas, and characterized by low energy efficiency, high pollution and underdeveloped renewables sectors (Aalto et al. 2017; Binhack and Tichý 2012). Most CEE countries have struggled to find the right balance between renewable, safe and diversified energy sources, while preventing mass unemployment through rapid de-carbonization. Hence, major energy interest organizations and labor unions may be crucial obstacles to or supporters of energy transitions.

Most CEE countries transformed their inherited communist health care model based on state ownership toward a national insurance authority or system of private insurers (Roberts 2009). Some CEE countries returned to pre-Soviet institutions based on the Bismarckian social insurance model. However, high debt forced many governments to introduce ‘out-of-pocket’ payments (Rechel and McKee 2009). This shift away from large state-run facilities was accompanied by measures to privatize and/or decentralize hospitals and services (Björkman and Nemec 2013). In view of these challenges and the heavy toll of coronavirus on CEE, effective channels for key health care stakeholders (patients, medical experts, health care workers) are more important than ever.

CEE higher education and science have also experienced processes of ‘simultaneous transition.’ Among the most crucial post-communist challenges were the dismantling of state planning and restoration of self-governance and academic freedoms, while achieving an effective balance between state regulation and institutional autonomy. International rankings have recently also shed light on the relative underperformance of CEE regarding research output, patents, and innovations. Therefore, governments have put forward targeted state strategies to promote university-industrial collaboration to generate “home-grown” human capital (Dobbins 2017). Student groups have strongly drawn on pre-existing traditions of student activism, which was crucial in toppling communism, and nowadays characterized by strong ideological heterogeneity in all four countries. University administrators have also formed interest associations to defend their interests vis-à-vis the state, while both student and rector organizations have become heavily interweaved with like-minded European-level organizations.

Altogether, our focus on three reform-intensive policy areas allows us to cover a sufficiently large pool of organizations for statistical analyses, while also enabling us to identify and focus on different group populations (e.g., medical patients, professional medical organizations; clean energy organizations, energy consumer groups, energy employees and suppliers; student organizations, professional academic organizations, see below). Yet this limitation to three policy areas also left us enough leeway for additional qualitative studies on the impact of (individual or multiple) interest organizations on specific policy processes (Horváthová and Dobbins 2019; Vlk et al. 2021; Labanino and Dobbins 2020). Hence, while previous international interest group surveys, notably the Comparative Interest Group Survey (Beyers et al. 2020) and INTEREURO survey (Beyers et al. 2014), operate with larger target populations,^2^ our project arguably has the advantage of zooming into the leverage of interest groups in specific policy areas and reform processes.

## The data collection process: population ecology

Analytically and empirically, the OrgIntCEE project is centered around five building blocks, each containing a bundle of key research questions tackled throughout the project: (1) population ecology, (2) interest intermediation, (3) access and influence of organized interests, (4) Europeanization and regionalization (5) and the ‘coming-of-age of organizations’ (for 2–5, see below). In addition to a series of interviews regarding specific reform processes, we compiled two datasets: a population ecology database and online survey (see supplementary data/online appendix for both datasets).

The backbone of the project is a population ecology dataset, which enabled us to apply and further develop existing approaches to population ecology (e.g., Hannan and Carroll 1992; Nownes 2004; Nownes and Lipinski 2005; Berkhout et al. 2015; Gray and Lowery 1996c). Mapping organizational populations is of outmost theoretical importance. Organizations are characterized by structural inertia stemming both from internal pressures (e.g., material and human resources, status quo bias within the organization) and external pressures (e.g., legal and fiscal barriers to entry or exit, cost of information, legitimacy constraints). Thus, their ability to adapt to environmental changes is limited (Hannan and Carroll 1992; Hannan and Freeman 1977, 1989). According to population ecology theory, organizational selection happens at the level of populations and not at the level of individual organizations. The question then is how changes in the organizational environment impact the ‘size distributions’ and ‘the diversity of organizational forms within broadly defined areas of activity.’ (Hannan and Freeman 1977: 957). Thus, the variation in founding rates and mortality rates of interest groups are functions of organizational (population) density (Hannan and Carroll 1992: 11). With the legitimation of an organizational form, the need for justification decreases, which reduces the cost of organizing. Ceteris paribus, the ‘legitimation of a form increases the founding rate of populations using that form, whereas competition induces a negative relationship between the density and founding rates’ (Hannan and Freeman 1989: 132–133). Hence, the number of organizations is constrained by ‘the availability of organizational resources, relatively independent of mobilization rates and dependent on the pre-existing density of organizations’ (Berkhout et al. 2015: 465).

Another compelling approach in population ecology research is the Energy-Stability-Area (ESA) model (Gray and Lowery 1996a, 1995, 1996c, 1996b; Lowery and Gray 1995), which also postulates that organizational diversity and density are affected by environmental—and not organization-level—variables. Environmental constraints, for example, are the number of potential members, the interests of constituents and issue certainty (= area term, or simply the ‘supply side’). Energy pertains to the ‘demand side,’ i.e., governmental actions and reforms with direct impact on interest groups, which are ‘vital resources interest organization entrepreneurs employ to secure sponsorship’ (Gray and Lowery 1996a: 106). The stability term is particularly relevant for CEE as it grasps profound disruptions to the political systems, such as (the end of) totalitarian rule or foreign occupation (Lowery and Gray 1995).

Against this background, we were interested in how organizational populations evolved in the communist and in particular post-communist phases. For example, how were pre-transition organizational populations impacted by different ‘varieties of communism’ (Labanino et al. 2021c) and how did the breakdown of communism (i.e., the ‘stability term’ of the ESA model) affect foundation rates? How did broader political reform processes and European integration, but also the recent trends toward illiberalism reshape organizational populations? And, critically, at what point did organizational populations become density-dependent (Hannan and Carroll 1992)? The dataset enables us to trace how various shifts in the political opportunity structure, i.e., political opening, political closure, but also media attention on related policy issues (Labanino et al. 2021a) drove organizational formations, as well as factors determining population density (Labanino et al. 2021b).

To define interest groups, we consistently applied Eising’s definition (2008) of three attributes inherent to interest groups: ‘organization, political interest, and informality’ (2008: 5). Organization means that they strive to ‘influence policy outcomes (…). Political interest refers to attempts (…) to push public policy in one direction or another on the behalf of constituencies or a general political idea,’ while ‘[i]nformality relates to the fact that interest groups do not normally seek public office but pursue their goals through informal interactions with politicians and bureaucrats’ (Eising 2008: 5).

### Population data sources

Compiling databases of our target populations based on these criteria posed significant, yet surmountable challenges. We applied a top-down mapping strategy for identifying relevant organizations (see, e.g., Fraussen et al. 2015; Hanegraaff et al. 2015). As a rule, following Rozbicka and Kamiński (2021), we collected data from public registries of civil society organizations. However, we built a dataset for organizations in three policy fields (see above) and active at the national level (i.e., we excluded regional and local organizations, but national umbrellas of subnational-level organizations). Another important difference is that we aimed at including all organizations that were active at any point in time between 1989 and 2018/2019, that is, dissolved or inactive ones as well. As we explain in detail below, this latter task was particularly difficult because of the shortcomings of the public records. That is, while we are certain that we captured all active organizations in 2018/2019 that fulfilled our selection criteria, the historical time-series data have to be used with keeping the serious data availability problems in mind (with a possible exception of Hungary, see below).

For Poland, we used the National Court Registry (*Krajowy Rejestr Sądowy*—KRS) as a starting point and were able to identify some additional organizations through the ngo.pl database. The KRS database unfortunately only indicates registrations from 2001, and all previously founded organizations are listed as being founded in 2001 (or 2002, 2003, i.e., the time of KRS registration). Therefore, we checked for each organization registered in this timeframe whether it was actually founded earlier and systematically searched for organizations founded before 2001.

Our main source for the Hungarian data was the Court Registry of Civil Society Organizations (*Civil szervezetek névjegyzéke*). The Hungarian court registry starts in 1989. Hence, organizations founded before nonetheless have 1989 as their founding date. In each case though, we checked for the actual foundation date. For Slovenia, our primary source was the AJPES registry (Agency of the Republic of Slovenia for Public Legal Records and Related Services—*Agencija Republike Slovenije za javnopravne evidence in storitve*). Our main source for the Czech data was the registry of the Czech Statistical Office (*Český statistický úřad*), while we also drew on the Systém IS NNO on the *Portál veřejné správy* (Public Administration Portal) to verify results: https://portal.gov.cz/

We used the same set of keywords to identify organizations in all four languages. First, across all three policy fields and in all four languages, we applied the different synonyms for different types of organizations (e.g., association, foundation, union, federation, etc., see online appendix). As in health care and higher education, there are highly specialized professional, patient, and student groups, and we also made a standardized list of medical professions (e.g., cardiology, pulmonology) and higher education disciplines^3^ to improve comparability (see online appendix). We cross-checked the identified organizations with Internet searches, lists from parliaments and ministries that invited organizations to various committees and interest intermediation bodies. We also reviewed secondary literature in the national languages to track down any organizations missing in the national registries.

Aside from Hungary, these registries do not contain information on dissolutions. Therefore, we systematically checked whether organizations were active or inactive with web searches and by contacting them. In many cases, we could not determine the exact date of dissolution, only the last date of activity or that the organization was active/inactive during data collection (2018–2020). As a rule, we determined any organization that does not have a website, Facebook site, or which has not been mentioned in any national newspaper articles in the past approx. three years to be inactive.

The information on dissolutions in the Hungarian registry also has limitations. The unified court registry was established in 2011, and the courts started to dissolve inactive organizations effectively as of 2014. As a result, there has been a “mass dissolution” of organizations ever since, even though most have been inactive for years (Sebestény 2017). We followed Sebestény (2017) in considering any organization as still existent, which was listed in the record as existent, did not exhibit any indications of a dissolution procedure (e.g., under liquidation), and had at least one report in the records since 2011.

Our experience showed that the four national registries are reliable, but imperfect sources of data and that each registry posed specific challenges. The Czech registry, for example, includes not only civil society organizations, but serves as a registry portal for businesses, making a clear distinction oftentimes difficult. The same holds for the Polish KRS—the search engine enables searches for businesses, foundations, associations, professional organizations, as well as health care facilities. While associations (*stowarzyszenia*) are mainly member-based, foundations (*fundacje*) belong to their founders and are a legal form of a non-governmental organization whose capital is allocated for a specific purpose. In the legal sense, a foundation is an asset (property money, securities, movables, real estate), which the founder may donate to achieve economically or socially important goals (e.g., education development, cancer prevention). Yet in many cases, foundations have staff, engage in political advocacy and effectively function as interest organizations. Therefore, we took a practical approach and included Polish foundations which verifiably are member-based, operate at the national level and represent collective interests to shape relevant policies (Berry and Wilcox 2018).

Gauging the higher education organization populations also posed some difficulties. For example, in some countries, i.e., Slovenia, universities themselves lobby governments and in much of the post-communist sphere *academies of science* are the main lobbying mouthpiece of the academic community. Academies of science were not included in the population ecologies as they are excluded from national registries but were surveyed due to their advocacy activities for the sector. Universities were only included in the database in the very few cases in which an invited organization (e.g., university workers’ association) filled out the survey using the name of a specific university, which is verifiably involved in university lobbying.

As mentioned, we only included organizations operating at the national level. We deem this limitation to be justified, as regionalization processes are a relatively new phenomenon in the sampled countries, and Hungary and Poland, in particular, have recently undergone processes of recentralization (Rozbicka et al. 2021b). This had the disadvantage of decreasing our sample size, but the advantage of filtering out countless organizations not predestined to be engaged in interactions with national political parties, ministries or European-level organizations. However, we did include organizations which originally had a local character but became national-level players over time (i.e., the environmental organization *Jihočeské matky*/Southern Czech Mothers).

We checked each organization’s website (or alternatively Facebook or Twitter site) not only for contact data, but also for information on founding dates, which may potentially contradict the data in the national registries. Table 1 provides a simple breakdown of our organizational population. Of the 1590 identified organizations, 1345 were active in 2018, and a total of 264 organizations in the sample were founded in or before 1989 (for organizations from the pre-communist and communist phase, see Labanino et al. 2021b).

**Table 1:** Breakdown of organizations by policy area and country

Policy area	Country				Total
	Czechia	Hungary	Poland	Slovenia	
Energy	158 (9.9%)	150 (9.4%)	135 (8.5%)	46 (2.8%)	489 (30.8%)
Health care	204 (12.8%)	188 (11.8%)	240 (15%)	171 (10.8%)	803 (50.5%)
Higher Education	47 (3%)	84 (5.3%)	86 (5.4%)	81 (5.1%)	298 (18.7%)
Total	409 (25.8%)	422 (26.5%)	461 (29%)	298 (18.8%)	1590

^*^Percentage of total sample in parentheses

### Coding of organizations

We then coded the organizations in each policy domain to smaller populations, which we classified as sub-fields and types (see dataset). Energy interest groups are divided into *fossil (and general)*, *nuclear*, *renewable* and *environmental protection* interest organizations. We only included environmental groups dealing specifically with clean energy and/or air pollution issues (i.e., environmental groups focusing on soil or animal protection were excluded). Higher education organizations are grouped based on whether they represent faculty/employee, student interests, institutional interests (e.g., rector’s conferences) or the interests of specific scientific communities (e.g., political science associations). Health care groups were sorted into five populations: business (e.g., pharmaceutical, medical devices), institutional (e.g., hospitals), non-medical employees, medical doctors and the medical professionals (including interest groups of respective medical professions), and patients. That is, we distinguished between 13 populations across three policy domains and thus a total of 52 national-level populations (see online appendix Table 1).

We also included a variable called subfield, which divides the different organizations to 13 sub-groups following a slightly different logic than our population variable (e.g., it has more detailed categories for health care groups—for example alternative, preventive—but only two categories for higher education groups). This provides another categorization alternative for researchers (see dataset, codebook). We additionally created an organizational type variable to grasp other organizational traits, namely whether they represent businesses, employees, employers, institutions, energy consumers, students, patients, medical doctors, etc. (see dataset, codebook). This makes it easier to pinpoint specific organizational sub-populations based on the constituents they represent, encompassing or specific character and other features. This breakdown of organizations also enabled us to identify any potential biases in response rate by organizational type. Regarding coding reliability, the original coding was carried out by project members with knowledge of the specific national language and policy areas after reviewing the organization’s website, if existent. The coding was then checked and re-checked by one of the project managers and another project member with knowledge of the country, language and policy area (see online appendix Table 1).

### Organizational formation rates and densities 1990–2018

**Fig. 1:**
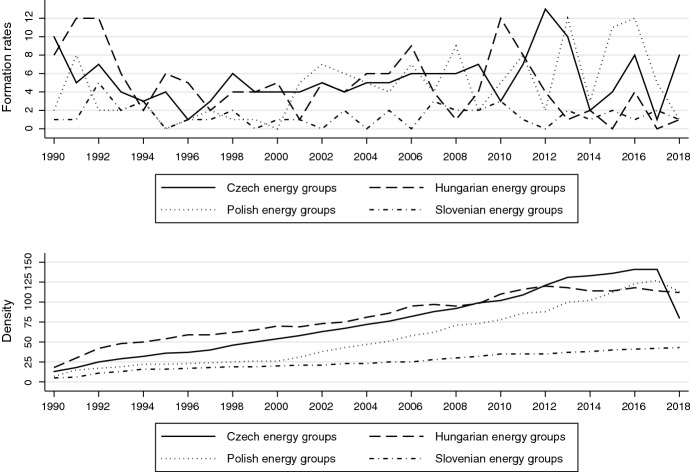
Energy policy organizations: formation and density rates: about here

Before discussing our survey, we concisely present some data reflecting organizational foundation rates since 1990, broken down by policy area. In energy policy (see Fig. 1), all four countries experienced a post-communist formation boom, followed by low formation activity in the mid-1990s. However, the run-up to EU accession (2004) again gave a strong boost to organizations. The graph also reflects the impact of the 2007 European Energy Strategy – and resulting national energy strategies (e.g., the 2007 “Energy Strategy until 2030” in Poland; the Czech State Energy Policy of 2015) on organizational formations.

Initially characterized by a smaller population, Slovenia also experienced a boom in foundations with the first (2008) and second (2014) National Energy Efficiency Action Plan. While Hungarian foundation rates initially mirrored those of the other countries, they have noticeably declined since 2011 under the national-conservative Orbán government in 2011, which may reflect a detrimental effect of democratic backsliding on organization foundations. The fact that the major National Energy Strategy adopted in 2012 (Horváthová & Dobbins, 2019) did not boost formations further supports this argument. It is also noticeable that population densities only seem to have recently plateaued in Czechia.^4^

**Fig. 2:**
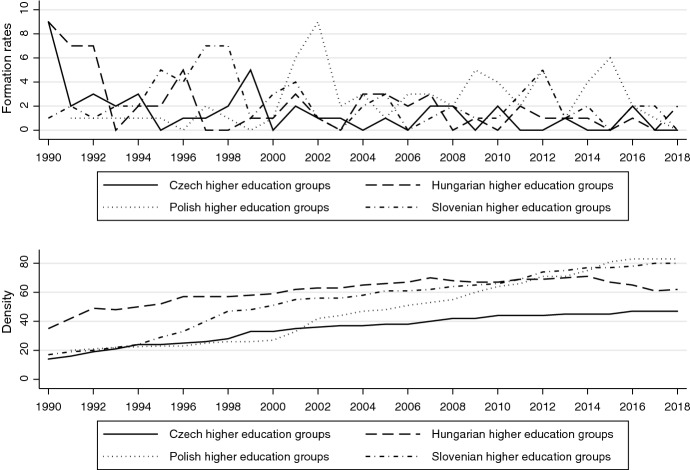
Higher education policy organizations: formation and density rates: about here

Regarding higher education (Fig. 2), we see similar developments and only slight signs of “saturation” in Hungary. The strong push to defend university autonomy and academic freedom after communism triggered a boom in student and academic organizations (e.g., rectors’ conferences), with Poland as a partial exception.

Then, we see another spike in formations in the run-up to or after the initiation of the Bologna Process in 1999, in Poland in particular. We see some moderate formation activity in 2010s, which can likely be attributed to the Polish government’s efforts to instill more market-oriented governance and stakeholder engagement (see Dobbins, 2015). Hungary started the 1990s with the highest number of organizations, peaking at 71 in 2014 and then declining to 62 in 2018. This lends some evidence to the observation by Labanino and Dobbins (2020) that civic activism against government encroachments on academic freedom has increasingly taken the form of social movements, rather than formal interest groups. Considering its very small higher education sector (four public universities), the large number of organizations in Slovenia also stands out. We would attribute this to the country’s strong tradition of (ideologically diverse) student democracy (Novak & Fink-Hafner 2019) and large number of professional academic organizations already existing under socialism.

**Fig. 3:**
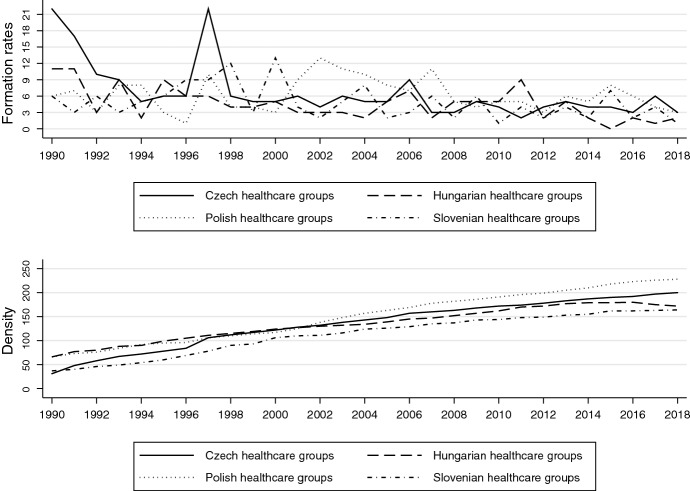
Health care organizations: formation and density rates: about here

A relatively large share of now active health care organizations (between 10 and 30%) were founded before 1990 (see Fig. 3). Hence, we only see a major immediate post-communist boom in Czechia, followed a few years later by a drastic spike in formations coinciding with the privatization of Czech health care (Kinkorová & Topolčan, 2012).

The first major post-communist reform in the late 1990s also boosted Polish the formation rates (Hellich & Wierzowiecka, 2017), which was followed by a “mini-boom” of patients’ organizations in particular around 2003, when a centralized National Health Fund was introduced. In Slovenia, by contrast, the switch to Bismarck-type social insurance and subsequent small-scale privatization measures only resulted in a handful of new foundations.

## A new comparative interest group survey

Constructing a targeted survey compatible with new lobbying systems, different policy areas and an extremely heterogeneous field of long-established and new organizations posed an enormous challenge. However, it offered numerous advantages over other forms of data gathering. First, the analysis of the so-called unobtrusive data sources (i.e., protocols of public consultations; e.g., Klüver 2009) is hardly feasible in CEE. Public databases of political consultations with interest groups have only recently emerged in many CEE countries, while in others, interest intermediation platforms have been dismantled (in particular Hungary, see Labanino 2020). Furthermore, unlike at the EU level, no online civic consultation platforms operate in our target countries. There is also a widespread observation that parliaments in democratic backsliding countries have become “law factories,” which quickly rubber-stamp laws (e.g., Várnagy 2012 for Hungary), making it even more difficult to gauge the clout of organized interests. Most importantly, if they exist, online consultation databases generally reveal little about how interest groups build coalitions, gain access, share expertise, develop internally and assess their influence.

These weaknesses can arguably be overcome with qualitative case studies. For example, process tracing (Beach and Pedersen 2019) is a very promising approach to studying specific reform processes, enabling us to test theoretical assumptions about interest group strength against empirical realities “on the ground.” However, process tracing, case study or interview-based interest group research (e.g., De Bruycker and Beyers 2019) demands enormous organizational and human efforts, spanning from identifying suitable reform process and involved actors, coordinating interviews and delving through archives, not to mention the objective analysis of gathered data. Furthermore, case studies tend to focus on major reform processes (Lowery 2013), while often neglecting smaller reforms or failed reform packages. Thus, process tracing may offer astute insights on developments of individual organizations (see, e.g., Fraussen 2014), but often cannot convey a sufficiently generalizable cross-country or cross-policy picture.

Therefore, much recent state-of-the-art interest group research has relied on large-scale (online) surveys (e.g., Beyers et al. 2020; Willems 2020). These bear the advantage of relatively low costs despite great human efforts involved in designing surveys, identifying target populations, generating responses and maintaining and data processing. Moreover, survey data offer a more suitable foundation for exploring the “coming-of-age” of interest organizations, i.e., level of professionalization, or the determinants of access to policy-makers. Against this background, we opted for a standardized survey with openings for additional input (e.g., optional open questions, comment function), while also carrying out case studies on various reforms in CEE (see Horváthová and Dobbins 2019; Vlk et al. 2021; Kubin 2021; Piotrowska 2020). As opposed to the CIG survey (Beyers et al. 2020), we did not apply a bottom-up sampling strategy, but relied on our population ecology dataset for the identification of the target population.

An English-language version of the survey was jointly developed by all project members and translated into the national languages. Particular attention was given to terminological clarity and the historical context. For example, the term “communist/post-communist” is frowned upon in Slovenia, while all in our four languages, the word “lobbying” carries a negative connotation. And, as mentioned above, there is still a widespread aversion to politics in the region, which compelled us to avoid political science jargon (e.g., corporatism, coalition-building, stakeholders, etc.).^5^

Considering the relatively small organization population size, generating an adequate response rate was a critical concern of the project consortium. The survey timeframe was from February 2019 to May 2020, which gave us enough time to contact and re-contact organizations. Essentially, we invited targeted organizations in different waves. Non-responding organizations received a first and second invitation reminder after approximately two weeks, respectively. We then waited several months before re-contacting repeatedly non-responsive organizations in the hope that changes in the staffing situation would facilitate response.

For nearly 100 of 1345 of the identified registered and supposedly active organizations in our population ecology dataset (see online appendix Table 1), we were unable to find contact data, resulting in a target population of 1247 organizations. Each contacted organization received an invitation email in the national language with a concise project description, accompanied by a personalized PDF invitation highlighting their key function in civil society.

Very specialized organizations perceived as less predestined to be involved in policy-making^6^ were contacted a total of three times by email. All organizations perceived as predestined to be influential (e.g., major energy unions, medical associations, student unions, etc.) were contacted up to five times, also by telephone.^7^

Online appendix tables 2, 3, 4 offer an overview of the response rate by country, policy area and sub-groups of organizations.

Altogether, we received responses from 436 of 1247 organizations, resulting in a total response rate of 34.9%, with 52% for Slovenia, 35.5% for Hungary, 33.8% for Czechia and 24.9% for Poland. The response rates pertain to all contacted organizations, including those less intensively contacted. We deem this a relatively significant achievement considering the small size of the project team, the instability of post-communist lobbying systems as well as the fact that our policy areas, in particular health care, cover many extremely specialized organizations (e.g., cytology). Our response rate also is roughly in line with that of the CIG project for CEE (see Rozbicka et al. 2021a).^8^ Importantly though, nearly all “major players” in the specific policy areas (e.g., large employers/employees unions, students unions, rectors’ conferences, large medical profession and patients associations) answered the survey due to the more intense contacting strategy. Hence, we are confident that the data (see online appendix/supplementary material) provide a solid grasp of the specific underlying lobbying dynamics.

### The logic and setup of the survey

**Fig. 4:**
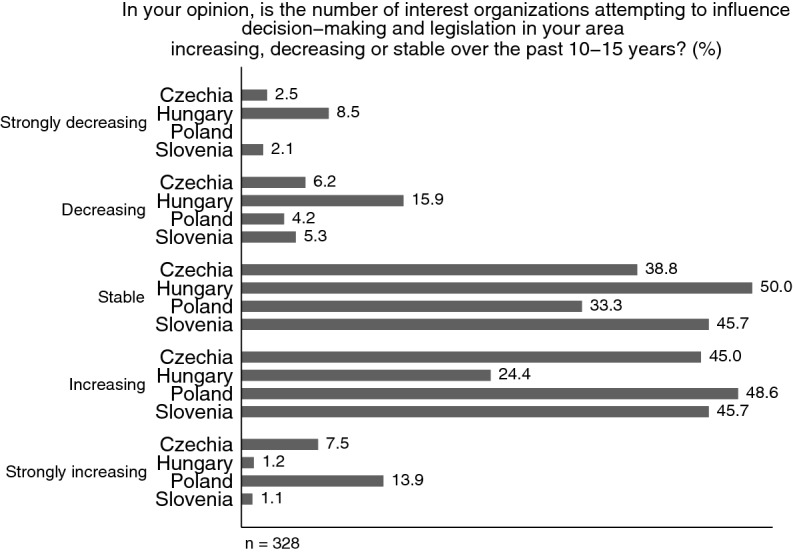
The change in perceived organizational densities in the respective field of activity compared to 10–15 years ago: about here

Aside from some general questions on membership (individuals, firms, institutions, volunteers, paid staff), funding (i.e., assessment of financial planning horizon) and national European, and international umbrella organization membership, we first aimed to generate insights on population ecology and density, in addition to the population databases (see above). We asked, among other things, about whether organizations sense an increase in other groups trying to influence legislation in their area and whether their own membership size has increased. In Fig. 4, we plotted the perceived densities per country. There is a clear variance in the sample across countries, whereas the proportion of those observing a decrease in organizations over the past 10–15 years is over 24% among Hungarian respondents, and it does not reach 10% in any of the other countries. Hungarian respondents perceive the highest stability (50%) followed by Slovenians (45.7%), whereas the corresponding numbers are less than 40% in both Poland (33.3%) and Czechia (38.8%). However, among those organizations reporting increasing or strongly increasing organizational densities, it is again Hungary, which is a clear outlier: Only 25.6% see growing group numbers compared to 47% of Slovenian, 52.5% Czech and 62.5% Polish respondents.

**Fig. 5:**
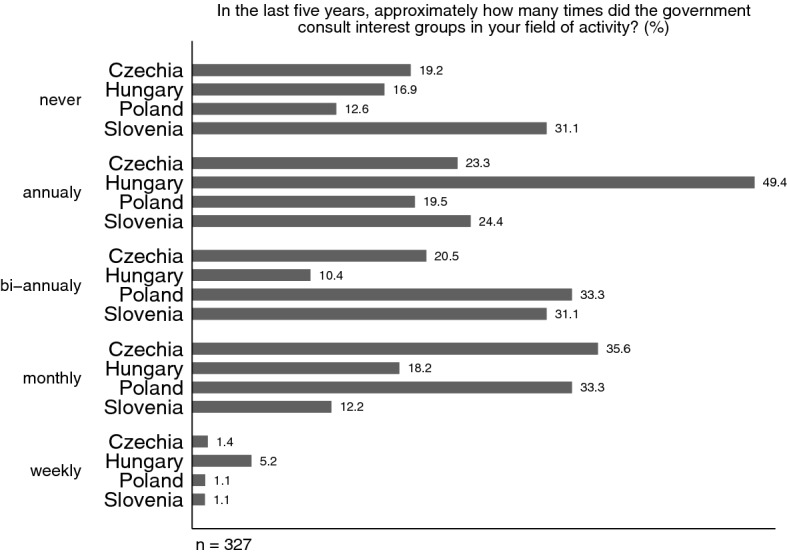
Perceived frequency of consultations with the government in the respective area of activity: about here

A second key analytical focus of the project is interest intermediation. We were interested in which political arenas organized interests operate (e.g., parliament, ministries/the executive, governing vs. opposition parties). Moreover, we were interested in whether lobbying patterns (Rasch 2018) have changed in the context of growing illiberalism and democratic backsliding. We asked, for example, *Approximately, how often does your organization consult with political parties / rivaling organizations / regulatory authorities?* (never– annually – bi-annually – monthly – weekly). As we have an array of questions about access to different policy-makers and venues (see below), here we asked organizations to give us a general evaluation on the frequency of governmental consultations with the government. *In the last five years, approximately how many times did the present / previous government consult interest groups in your field of activity?* (never– annually – bi-annually – monthly – weekly).^9^ This enabled us to also measure the perceived openness of the political opportunity structure at the organizational level rather than merely individual access to policy-makers. We plotted the distribution of responses per country for the frequency of consultations in the policy field with the current government (Fig. 5). The graph shows very interesting country-specific differences. 56.1% of Czech and a whooping 66.6% of Polish groups report government consultations twice a year or monthly in their area of activity. The corresponding proportions are 43.3% for Slovenian and only 28.6% for Hungarian respondents. Almost a third of Slovenian groups contend that there are no government consultations in their field of activity, which is by far the highest proportion. This might be due to the highly corporatist structure of Slovenian interest representation. It is in turn in Hungary, where annual consultations are the most common answer (49.4%). Interestingly, weekly consultations are also reported in the highest proportion in Hungary (5.2%), which may indicate that a few selected groups enjoy almost barrier-free access to policy-makers (see Horváthová and Dobbins 2019 for precisely this phenomenon in the Hungarian energy sector).

An additional analytical anchor for this bundle of questions is the classic distinction between corporatism, pluralism, and statism. The responses enable us to assess to what extent post-communist interest intermediation systems gravitated toward one of these ideal-types (e.g., for health care, see Dobbins et al. 2021; for energy policy, see Horváthová et al. 2021). While much previous research on corporatism and indicators of it relies on observations and data gathered from it (e.g., Jahn 2016; Siaroff 1999), the data allow us to derive corporatism scores based on data directly from interest groups themselves, for example regarding their level of policy coordination with the state and the consensual orientation of policy-making (see survey in online appendix).

**Fig. 6:**
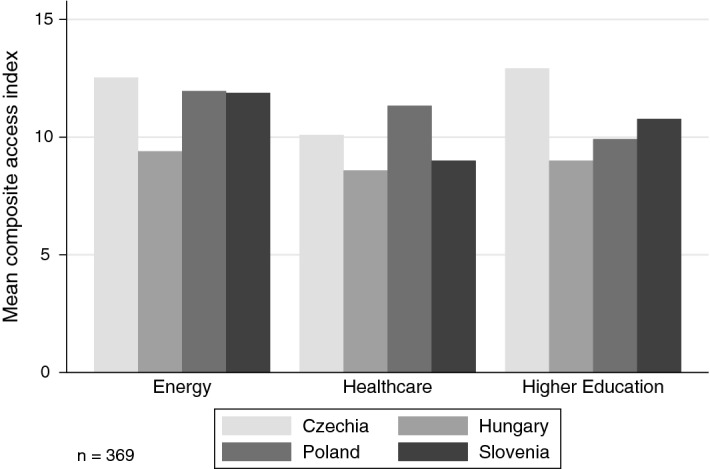
Mean composite access index per policy field and country: about here

Our third building block addresses access and influence. A key measure of political inclusion is to what extent groups can access policy-makers and different policy-making venues (Binderkrantz et al. 2014, 2015; Eising 2007), which is an imperative (albeit an unnecessarily sufficient condition) for influence. We therefore asked about access to governing parties, regulatory authorities, opposition parties, parliaments, as well as a general evaluation of the level of policy coordination with the state. If we combine these five survey items—all measured on a 5-point scale each—we get a “composite access” index ranging potentially from 1 to 25. Figure 6 reveals consistent differences in access across countries and policy fields. Generally, energy policy organizations enjoy the highest access (except for Czech higher education groups, which report the easiest access), whereas somewhat surprisingly, health care organizations the least. Hungarian organizations have the lowest access across all three policy fields, whereas Polish democratic backsliding does not seem to limit access (at least in these policy fields).

**Fig. 7:**
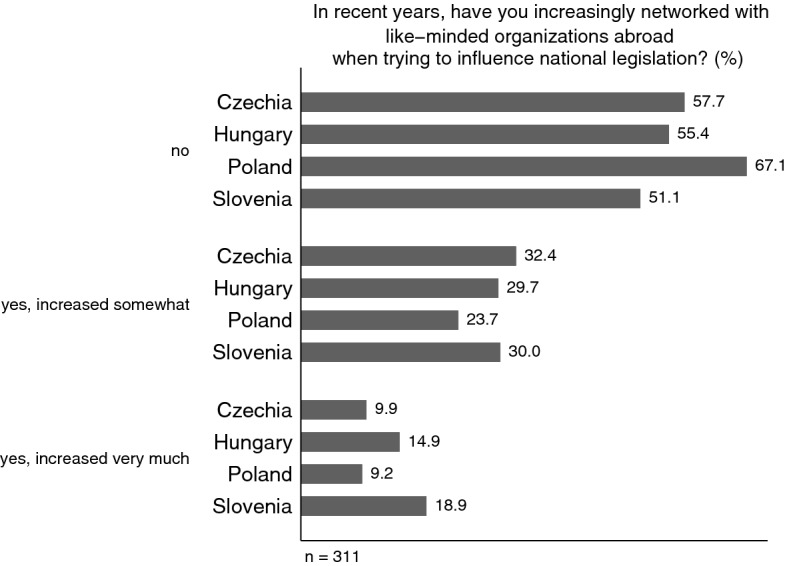
International networking as a means of influencing national policy-making: about here

Considering the monumental impact of **Europeanization** on CEE, we were also interested in how engagement with European-level and international organizations abroad affects CEE organized interests. Europeanization opened a new world of transnational lobbying opportunities, and it is plausible that organizations with difficulties accessing national political institutions may flee to the European level—and potentially ‘bring back home’ expertise and skills gained there. Aside from questions on umbrella affiliations, we inquired about existing and increasing transnational ties, various types of support from organizations abroad as well as their concrete impact. For example, a question about strong ties with like-minded organizations in other EU countries revealed that it is by far Slovenian groups that have an international network: 76.8% compared to 40–50% in the other three countries. However, if we asked whether they used their international network to influence national policy-making, we get a much more interesting picture (Fig. 7). It is Poland, where the share of groups that indeed use international networking to influence national policy outcomes is the lowest (32.9%), whereas in Slovenia (48.9%), Hungary (44.6%) and in Czechia (42.3%), it is significantly higher.

As hinted above, we finally focus on the “coming-of-age” dimension. In new democracies, organizational professionalization may be an additional key to democratization, as the more professionalized organizations are, the more effectively they defend the interests of constituents. Seen critically though, organizations with professionalized lobbying staff may also become detached from their member base (Hwang and Powell 2009; Heylen et al. 2020). In line with established conceptions of professionalization (e.g., Klüver and Saurugger 2013; Imig and Tarrow 2001), we asked, for example, whether organizations are focusing on internal and human resource development, fundraising, strategic planning, the training of lobbyists and the evaluation of efficiency and effectiveness compared to 10–15 years ago (measured on 5-point scales ranging from much less to much more).

**Fig. 8:**
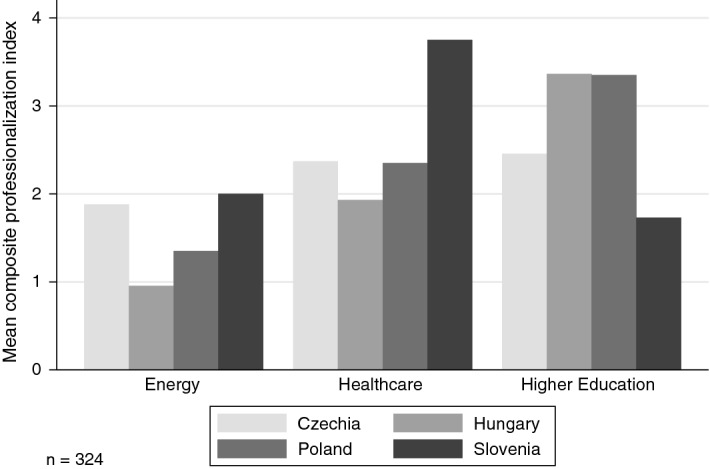
Level of professionalization as compared to 10–15 years ago: about here

In Fig. 8, we plotted the mean total composite professionalization index (a sum of all six items). We recoded the six items so that a decreasing focus on an area takes a negative sign, whereas increasing focus takes a positive one and no change is coded 0. This way we get a composite variable ranging from −12 to + 12. As the bar charts show, organizations across policy fields in all four countries have become on average more professionalized over the last 10–15 years. Energy groups, particularly in Hungary and Poland, invested the least in enhancing their operations, whereas health care and particularly higher education groups have become increasingly professionalized.

**Fig. 9:**
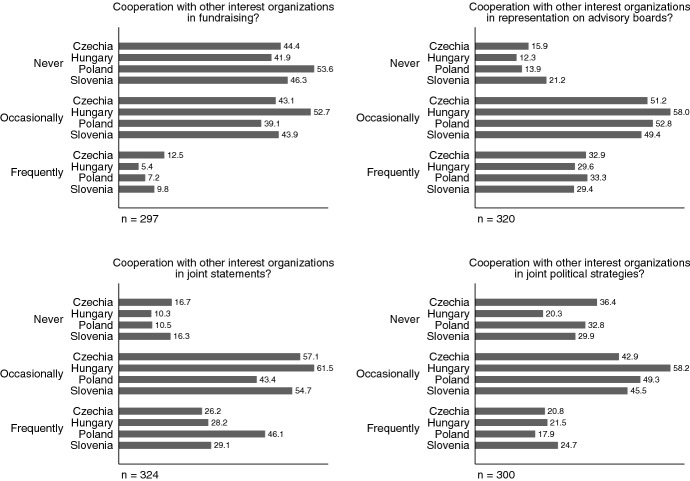
Modes of domestic intergroup cooperation: about here

Regarding inter-organizational cooperation, which is broadly recognized as an important factor boosting interest groups’ influence (Mahoney & Baumgartner, 2015; Junk 2019), we also asked about organizations’ ability to cooperate with others in various ways. Intergroup cooperation is particularly important in the context of relatively weak civil societies and democratic backsliding as it is one of the most important strategies against organizational failure (Pfeffer and Salancik 2003; Hanegraaff and Pritoni 2019). We asked our respondents to indicate if they cooperate (and if yes, whether occasionally or frequently) with other groups in four important areas: fundraising, representation on advisory boards, the issuing of joint statements and joint political strategies. Indeed, as shown in Fig. 9, organizations value cooperation with other groups. Yet as the previous figures show, Hungarian groups evaluate the political opportunity structure the least open in general and report the least individual access to policy-makers, too. It is therefore not surprising that the share of Hungarian groups reporting no cooperation is consistently the smallest in all four areas. This provides evidence for the importance of intergroup cooperation in an increasingly hostile political environment for organized interests.

The data generated from this block can be used as both dependent and independent variables, and neatly linked to the other four dimensions. For example, how do organizational population densities affect professionalization or inter-organizational cooperation? How does European-level engagement or international umbrella organization membership affect organizational professionalization? Does being involved in corporatist-style arrangements drive organizational professionalization and inter-organizational cooperation? And as hinted above, the data enable the creation of organization-specific professionalization indicators, so that we can assess their impact on political access, influence and involvement in political consultations.

## Lessons drawn and the path forward

Despite the generous funding, the constraints of the project regarding duration (initially three years) and staff as well as its analytical multidimensionality combined with the multi-methods approach placed various constraints on the scope of the survey. The project team members from diverse academic cultures had to find relatively quick consensus on a uniform catalog of survey questions and digital modus operandi viable across four strikingly different political systems and three policy areas.

Therefore, numerous compromises were made and, retrospectively, the project team agrees that various things could have been done somewhat differently. First, at a practical level, more pretests should have been conducted. Even though the survey was translated professionally by native speakers, various terminological issues slightly depressed the response rate for certain questions. For example, many organizations were unfamiliar with the term “umbrella organization,” while others had difficulties with concepts such as “opportunity structures.” In fact, numerous organizations did not even view themselves as political or predestined to engage with policy-makers and/or other interest groups in their members’ interest. Some organizations refrained from answering questions about policy coordination with the state, as public advocacy was not perceived as a core organizational activity.

Nevertheless, we generated a relatively high response rate and wealth of quantitative data. The many additional comments (over 1000) from respondents were further proof that the survey was, by and large, well-received and offer insightful nuances which quantitative data cannot capture. However, a further weakness of our survey is arguably its overfocus on interest groups’ engagement with the state, parties and governmental institutions, i.e., inside lobbying strategies. Self-critically, we should have delved deeper into organizations’ outside, indirect lobbying activities as there are growing indications that civil societies organizations in ‘backsliding’ countries are disengaging from government policy-makers and taking their activities ‘to the streets’ (for Hungary, see Labanino and Dobbins 2020). Despite the growing importance of media activities by interest groups (Binderkrantz et al. 2015), our dataset did not cover this aspect.^10^ Another overlooked dimension, which lends itself to future exploration, is the relationship between members and organizational leadership. To what extent are post-communist organizations governed by their members? Are internal decision-making mechanisms more bottom-up or top-down?

Finally, various developments took place throughout the project, most notably the Covid pandemic (2020 +). Not only did it challenge project logistics, but the pandemic-related restrictions heavily affected all three policy areas. Therefore, one route for further research is to explore the evolution of advocacy activities not only amid pandemic-related digitalization, but also the associated decline of democratic standards, particularly in Hungary and Poland. Moreover, the limitation to only four CEE countries means that we have neglected the notoriously understudied Balkan region and its organizational landscapes as well as Eastern European countries more heavily affected by Russian dominance and/or aggression such as Ukraine, Armenia and Moldova. Clearly, the much-desired return to peace, post-war recovery, and reawakening of civil society in Ukraine will create new opportunities for interest group researchers. The lessons drawn from the OrgIntCEE project therefore will hopefully encourage researchers to push the geographical focus further eastward, while also tackling new policy areas directly affected by the war (most prominently defense and security policy, but also housing, agriculture, tourism, and transportation). Altogether, there is still a huge demand for further investigation of other sectors and in-depth comparisons with the previously examined policy areas.

The scientific utility of the dataset (see online appendix/supplementary material) does not end with the completion of the project, rather will hopefully serve as a point of reference for many further inquiries. Therefore, the authors invite all interested scholars to engage with the collected data to enhance the scholarly exploration of civil society and organized interests in a complex and unpredictable, but always fascinating region.

## Supplementary Information

Below is the link to the electronic supplementary material.

Supplementary file1 (DOCX 16 KB)

Supplementary file2 (DOCX 15 KB)

Supplementary file3 (DOCX 15 KB)

Supplementary file4 (DOCX 21 KB)

Supplementary file5 (DOCX 13 KB)

Supplementary file6 (DOCX 17 KB)

Supplementary file7 (DOCX 13 KB)

Supplementary file8 (DOCX 13 KB)

Supplementary file9 (DOCX 77 KB)

Supplementary file 10 (PDF 1078 KB)

Supplementary file 11 (XLSX 395 KB)

Supplementary file 12 (DTA 12311 KB)

Supplementary file 13 (XLSX 148 KB)

Supplementary file 14 (DTA 809 KB)

## Footnotes

1 The project has been generously funded by the *Deutsche Forschungsgemeinschaft* (DFG—German Research Foundation) and *Narodowe Centrum Nauki* (NCN—National Science Center) and carried out by a multinational team of scholars. The order of authors is based on length of involvement in the project. All authors contributed equally to data collection and compiling the survey dataset (see online appendix/supplementary files).

2 And in the case of INTEREURO a set of 20 EU directive proposals.

3 For higher education, we used Wikipedia’s outline of academic disciplines: https://en.wikipedia.org/wiki/Outline_of_academic_disciplines; for example, we included historical or philosophical associations, but not specific organizations such as “The Association of Eastern European Historians.” Moreover, we narrowed down the population to organizations with active university personnel and mentions of HE and science policy on their website.

4 The sharp decline in Czech energy organization density in 2018 reflects data quality. While we were able to determine that a number of organizations have become inactive or were dissolved during the 2010s, we were unable to pinpoint the exact dates of their dissolution.

5 All survey participants received essentially identical survey questions, aside from some very small country- and policy-specific nuances, e.g., references to currently and previously governing parties, references to regulatory authorities in specific policy areas.

6 As a fictive example: the Association of Latvian Students in Poland.

7 Toward the end of the survey period, we successfully re-contacted a few organizations perceived as absolutely critical (e.g., coal mining unions, rectors’ organizations) by telephone with the offer to complete an ‘express’ 3-min online or telephone version of the survey with just 8–10 questions essential for individual project-related papers.

8 Lithuania—41%, Poland 28%, Slovenia 36%.

9 We subsequently asked respondents how often they participated on these meetings. This was unfortunately an open question, so we received a lot of “as often as we were invited” type of answers, which are very difficult to quantify.

10 For a spin-off project covering inside/outside lobbying in CEE, see Czarnecki (2021).
