# Supplementary material for: Organized interests in post-communist policy-making: a new dataset for comparative research
Source: Interest Groups Advocacy. 2022 Nov 15;12(1):73–101. doi: 10.1057/s41309-022-00172-1 (PMC9665044; doi:10.1057/s41309-022-00172-1)
Supplement: Supplementary file 1 — Supplementary file1 (DOCX 16 KB) [file 41309_2022_172_MOESM1_ESM.docx]

**Appendix Table 1: Country- and policy-specific populations with sub-types**

|  |  | | CZ  2018 | CZ  1989 | HU  2018 | HU  1989 | PL  2018 | PL  1989 | SLO  2018 | SLO  1989 |
| --- | --- | --- | --- | --- | --- | --- | --- | --- | --- | --- |
| Healthcare  groups | HC business | | 12 | 0 | 6 | 1 | 8 | 0 | 1 | 0 |
|  | HC institutions | | 18 | 0 | 5 | 1 | 12 | 0 | 22 | 10 |
|  | Medical professionals | | 68 | 14 | 124 | 47 | 139 | 50 | 67 | 13 |
|  | Non-medical employees | | 8 | 0 | 6 | 2 | 15 | 2 | 13 | 1 |
|  | Patients | | 92 | 5 | 29 | 6 | 49 | 6 | 60 | 8 |
| Higher education groups | HE scientific | | 19 | 10 | 30 | 15 | 37 | 12 | 29 | 12 |
|  | HE students | | 12 | 1 | 17 | 5 | 38 | 5 | 44 | 4 |
|  | HE institutions | | 7 | 0 | 8 | 3 | 8 | 1 | 1 | 0 |
|  | HE employees | | 3 | 0 | 8 | 6 | 2 | 2 | 4 | 1 |
| Energy policy  groups | Fossil energy | | 36 | 1 | 48 | 8 | 31 | 3 | 13 | 1 |
|  | Renewable energy | | 19 | 0 | 30 | 0 | 56 | 0 | 10 | 2 |
|  | Environmental protection | | 21 | 2 | 7 | 2 | 22 | 2 | 16 | 0 |
|  | Nuclear energy | | 4 | 0 | 6 | 0 | 3 | 0 | 2 | 0 |
|  | Country totals | | 319 | 33 | 324 | 96 | 420 | 83 | 282 | 52 |
|  | Sample total | |  |  |  |  |  |  |  |  |
|  | **2018: 1345** | **1989: 264** |  |  |  |  |  |  |  |  |
