# Supplementary material for: Organized interests in post-communist policy-making: a new dataset for comparative research
Source: Interest Groups Advocacy. 2022 Nov 15;12(1):73–101. doi: 10.1057/s41309-022-00172-1 (PMC9665044; doi:10.1057/s41309-022-00172-1)
Supplement: Supplementary file 2 — Supplementary file2 (DOCX 15 KB) [file 41309_2022_172_MOESM2_ESM.docx]

**Appendix Table 2: Responding healthcare organizations by country and type**

| **Healthcare** | | | | |
| --- | --- | --- | --- | --- |
| **Country** | **Responses** | **% of total responses** | **Invited** | **Response rate per country/type in country** |
| **Czechia – Total** | **68** | **31.3%** | **214** | **31.7%** |
| Med. profession | 16 |  | 73 | 21.9 % |
| Patients | 42 |  | 99 | 42.2 % |
| Hospitals/Institutional | 2 |  | 9 | 22.2 % |
| Business/Pharma | 5 |  | 21 | 23.9 % |
| Employees | 3 |  | 12 | 25.00 % |
|  |  |  |  |  |
| **Hungary – Total** | **53** | **24.4%** | **145** | **36.5%** |
| Med. profession | 23 |  | 83 | 27.7 % |
| Patients | 22 |  | 43 | 51.2 % |
| Hospitals | 0 |  | 3 | 0 % |
| Business/Pharma | 4 |  | 8 | 50 % |
| Employees | 4 |  | 8 | 50 % |
|  |  |  |  |  |
| **Poland - Total** | **49** | **21.2%** | **193** | **25.4 %** |
| Med. profession | 13 |  | 125 | 10.4 % |
| Patients | 24 |  | 43 | 55.8 % |
| Hospitals/Institutional | 2 |  | 6 | 33.3 % |
| Business/Pharma | 3 |  | 8 | 37.5 % |
| Employees | 2 |  | 2 | 100% |
| Employers | 5 |  | 9 | 55.6% |
|  |  |  |  |  |
| **Slovenia – Total** | **50** | **22.7 %** | **160** | **31.3%** |
| Med. profession | 11 |  | 70 | 14.3 % |
| Patients | 29 |  | 59 | 49.2% |
| Hospitals/Institutional | n.a. |  | n.a. | n.a. |
| Business/Pharma | 2 |  | 3 | 66.7 % |
| Employees | 7 |  | 9 | 77.8 % |
| Employers | 1 |  | 1 | 100 % |
| **Total – All Countries** | **220** |  | **712** | **30.89%** |
