# Supplementary material for: Organized interests in post-communist policy-making: a new dataset for comparative research
Source: Interest Groups Advocacy. 2022 Nov 15;12(1):73–101. doi: 10.1057/s41309-022-00172-1 (PMC9665044; doi:10.1057/s41309-022-00172-1)
Supplement: Supplementary file 3 — Supplementary file3 (DOCX 15 KB) [file 41309_2022_172_MOESM3_ESM.docx]

**Appendix Table 3 Responding higher education organizations by country and type**

| **Higher Education** | | | | |
| --- | --- | --- | --- | --- |
| **Country** | **Responses** | **% of total responses** | **Invited** | **Response rate per country/type in country** |
| **Czechia – Total** | **16** | **15.2 %** | **48** | **33.3 %** |
| Rectors - institutional | 3 |  | 8 | 37.5 % |
| Acad. profession | 6 |  | 20 | 30 % |
| Students | 6 |  | 16 | 37.5 % |
| HE employees | 1 |  | 4 | 25 % |
| Educational promotion | n.a. |  | n.a |  |
|  |  |  |  |  |
| **Hungary - Total** | **17** | **16.2 %** | **49** | **34.7 %** |
| Rectors - institutional | 2 |  | 4 | 50 % |
| Acad. profession | 8 |  | 22 | 36.3 % |
| Students | 4 |  | 13 | 30.7 % |
| HE employees | 3 |  | 8 | 37.5 % |
| Educational promotion | n.a. |  | n.a. |  |
| **Poland – Total** | **25** | **33.3 %** | **65** | **38.5 %** |
| Rectors institutional | 3 |  | 4 | 75 % |
| Acad. profession | 6 |  | 9 | 66.7 % |
| Students | 8 |  | 27 | 29.7 % |
| HE employees | 1 |  | 1 | 100 % |
| Educational promotion (incl. foundations) | 7 |  | 24 | 29.1 % |
|  |  |  |  |  |
| **Slovenia – Total** | **37** | **35.2 %** | **64** | **57.8%** |
| Rectors/institutional | 1 |  | 1 | 100 % |
| Acac. profession | 13 |  | 20 | 65 % |
| Students | 16 |  | 35 | 45.7% |
| HE employees | 5 |  | 5 | 100 % |
| Educational promotion | 2 |  | 3 | 66.6 % |
|  |  |  |  |  |
| **Total – all countries** | **95** |  | **226** | **42 %** |
