# Supplementary material for: Organized interests in post-communist policy-making: a new dataset for comparative research
Source: Interest Groups Advocacy. 2022 Nov 15;12(1):73–101. doi: 10.1057/s41309-022-00172-1 (PMC9665044; doi:10.1057/s41309-022-00172-1)
Supplement: Supplementary file 4 — Supplementary file4 (DOCX 21 KB) [file 41309_2022_172_MOESM4_ESM.docx]

**Appendix Table 4 Responding energy organizations by type^^[[1]](#footnote-1)^^**

| Country | responses | % of total responses | Invited | response rate in % |
| --- | --- | --- | --- | --- |
| Czechia – Total | **37** | **31.4%** | **106** | **34.9%** |
| Nuclear/Fossil | 7 |  | 17 | 41.2% |
| Renewables | 10 |  | 29 | 34.5% |
| Env. protection | 10 |  | 26 | 38.5% |
| Employers/firms | 16 |  | 30 | 53.3% |
| Employees | 6 |  | 10 | 60% |
|  |  |  |  |  |
| Hungary – Total | **26** | **22%** | **79** | **33%** |
| Nuclear/Fossil | 10 |  | 12 | 83.3% |
| Renewables | 8 |  | 29 | 27.6% |
| Env. protection | 3 |  | 7 | 42.8% |
| Employers/firms | 7 |  | 14 | 50% |
| Employees | 6 |  | 15 | 40% |
|  |  |  |  |  |
| Poland – Total | **28** | **21.2%** | **78** | **35.9%** |
| Nuclear/Fossil | 4 |  | 11 | 36.7% |
| Renewables | 8 |  | 25 | 32% |
| Env. protection | 6 |  | 14 | 42.8% |
| Employers/firms | 12 |  | 28 | 42.9% |
| Employees | 2 |  | 6 | 33.3% |
|  |  |  |  |  |
| Slovenia – Total | **30** | **25.4%** | **46** | **65.2%** |
| Nuclear/Fossil | 4 |  | 6 | 66.7% |
| Renewables | 5 |  | 9 | 55.6% |
| Env. protection | 16 |  | 22 | 72.7% |
| Employers | 7 |  | 10 | 71% |
| Employees | 10 |  | 14 | 71% |
|  |  |  |  |  |
| Total – all countries | **121** |  | **309** | **39.1%** |

1. For clarity, the table allows for double classifications (e.g., nuclear energy/renewables **and** employers or employees). Therefore, the numbers of invited and responding organizations by organizational type are higher than the total numbers. [↑](#footnote-ref-1)
