# Supplementary material for: Organized interests in post-communist policy-making: a new dataset for comparative research
Source: Interest Groups Advocacy. 2022 Nov 15;12(1):73–101. doi: 10.1057/s41309-022-00172-1 (PMC9665044; doi:10.1057/s41309-022-00172-1)
Supplement: Supplementary file 5 — Supplementary file5 (DOCX 13 KB) [file 41309_2022_172_MOESM5_ESM.docx]

**List of search terms: energy policy**

Accumulation

Biodiesel

Biomass

Chemical (energy, industry)

Climate

Coal

Conservation

Earth

Ecology / ecological / eco

Electricity / electricians

(clean, safe, secure, alternative, independent) Energy

Energy (consumers, producers, distributors, employers, employees, industry)

Environment

Gas

Green

Heat/heating

Hydro(power) / water energy

Hydraulic

Mine/mining

Nuclear

Oil

Petroleum

Photovoltaic

Pipeline

Planet

Power (industry)

Protection

Renewable

Shale gas

Solar

Sustainable

Thermal

Waste

Wind
