# Supplementary material for: Organized interests in post-communist policy-making: a new dataset for comparative research
Source: Interest Groups Advocacy. 2022 Nov 15;12(1):73–101. doi: 10.1057/s41309-022-00172-1 (PMC9665044; doi:10.1057/s41309-022-00172-1)
Supplement: Supplementary file 6 — Supplementary file6 (DOCX 17 KB) [file 41309_2022_172_MOESM6_ESM.docx]

**List of search terms: healthcare**

Acupuncture

Addictology

Aesthetic Medicine / Anti-Aging

AIDS (support)

Allerology/Asthma

Alzheimer

Angiology

Andrology

Anesthesiology

Artery

Autism

Biomedicine

Blood

Brain / Cerebral damage

Breast cancer

Cancer

Cardiology

Cerebral –

Chron

Clinical nutrition

Clinic(al)

Colon cancer / proctology

Cystic fibrosis

Cytology

Dentistry/stomatology

Diabetes

Dialtransplant / Dialysis / Kidney Transplant

Diatetics

Dementia

Disability / disabled

Doctor

Drug abuse

Emergency doctors / physicians / medical services

Endodontics

Endoctrinology

Epidemiology

Epileptology

Eye doctors / surgeons

Family medicine

Gastroenterology

General medicine

Geriatrics

Gynecology

Headache

Healthcare

Healthcare management

Heart

Hematology

Hemophilia

Hepatology / liver

Homeopathy

Hospitals

(Human) genetics

Huntington’s disease

Hygiene

Hypertension

Immunology

Infectious disease

Internists, internal medicine

Implantology

Laryngology

Leukemia

Logopedia / Speech therapy

Lung

Lymphoma

Managed Care/ Health Care Managers

Medicine/medical

Menopause

Mental health

Metabolic

Midwives

Multiple sclerosis

Muscular dystrophy

Naturopathy

Nephrology

Neonatology

Neuro-

Neuroendocrinology

Neurology

Neurophysiology

Neurosurgery

Nuclear medicine

Nurses

Obesity

Occupational medicine

Oncology

Opticians/Optometry

Orthodonic

Orthopedia

Osteoporosis

Otorhinolaryngologists, Phoniatrists and Audiologists (head, neck, ears)

Outpatient/Ambulant

Palliative (care)

Patient

Pathology

Parkinson’s

Pediatrics

People with disabilities

Periodontolgy

Pharmaceutics

Pharmacists

Physiotherapy

Plastic surgery

Podology / foot doctors

Prevention

Practitioner

Prostate Diseases

Psychiatry / Psychotherapy

Psychology

Psycho-oncology

Pulmonology / Lung diseases

Radiology

Rare diseases

Red Cross

Rehabilitative / rehabilitation

Residence/Doctors in residence

Respiratory medicine

Rett syndrome

Rheumatology

Sclerosis

Sexology

Sleep medicine

Sociotherapy

Spine – spinal surgery

Stomatology/ Dentistry

Stroke

Surgery

Tourette Syndrome

Traumatology

Transplantology

Thyreologie / Thyroid

Urogynaecology

Urology

Vascular diseases

Venerology

Virology

(In) Vitro
