# Supplementary material for: Organized interests in post-communist policy-making: a new dataset for comparative research
Source: Interest Groups Advocacy. 2022 Nov 15;12(1):73–101. doi: 10.1057/s41309-022-00172-1 (PMC9665044; doi:10.1057/s41309-022-00172-1)
Supplement: Supplementary file 7 — Supplementary file7 (DOCX 13 KB) [file 41309_2022_172_MOESM7_ESM.docx]

**List of search terms: Higher education policy**

Academic/academics

Anthropology

Applied Sciences

Archeology

Biology

Chemistry

College

Computer science/informatics

Economics

Faculty

Geography

Graduate

Higher education

History

Intellectual

Languages/linguistics

Law

Mathematics

Non-compulsory education

Philosophy

Physics

Political Science

Psychology

Rector

Research

Science

Sociology

Student

Tertiary education

Theology

University

**Note:** For professional scientific associations, we used Wikipedia’s outline of academic disciplines: <https://en.wikipedia.org/wiki/Outline_of_academic_disciplines> (as of December 2020); for example, we included historical or philosophical associations, but not specific organizations such as “The Association of Eastern European Historians”. Moreover, we narrowed down the population to organizations with active university personnel and mentions of HE and science policy on their website. By contrast, all student associations – regardless of the discipline and its specificity – were included.
