# Supplementary material for: Organized interests in post-communist policy-making: a new dataset for comparative research
Source: Interest Groups Advocacy. 2022 Nov 15;12(1):73–101. doi: 10.1057/s41309-022-00172-1 (PMC9665044; doi:10.1057/s41309-022-00172-1)
Supplement: Supplementary file 8 — Supplementary file8 (DOCX 13 KB) [file 41309_2022_172_MOESM8_ESM.docx]

**Table 5 – List of terms for association, organization, etc.**

| **Czech** | **Polish** | **Hungarian** | **Slovene** |
| --- | --- | --- | --- |
| Asociace | Stowarzyszenie | Társaság | Združenje |
| Svaz | Towarzystwo | Szövetség | Društvo |
| Družstvo | Zrzeszenie | Egyesület | Zveza |
| společnost | Federacja | Szakszervezet | Sindikat |
| sdružení | Fundacja | Federáció | Ustanova |
| unie | Reprezentacja | Konfederáció | Konfederacija |
| Spolek | Korporacja | Képviselet | Zbornica |
| Aliance | Unia | Alapítvány | fundacija |
| Organizace | Związek | Szervezet |  |
|  | Izba | Tanács |  |
|  | Organizacja | Önkormányzat |  |
|  | Liga | Kamara |  |
|  |  | Liga |  |
