# Supplementary material for: Organized interests in post-communist policy-making: a new dataset for comparative research
Source: Interest Groups Advocacy. 2022 Nov 15;12(1):73–101. doi: 10.1057/s41309-022-00172-1 (PMC9665044; doi:10.1057/s41309-022-00172-1)
Supplement: Supplementary file 9 — Supplementary file9 (DOCX 77 KB) [file 41309_2022_172_MOESM9_ESM.docx]

Appendix

Overview of the OrgIntCEE survey

Survey Languages: Czech, English, Hungarian, Polish, Slovenian

Survey Timeframe: February 2019–June 2020

Survey Platform: LimeSurvey

Survey Questions

| What is the name of your organization? |
| --- |
| Does your organization represent business interests? Yes/No |
| Are you an umbrella organization? Yes/No |
| How many individuals are members of your organization? |
| How many firms are members of your organization? |
| How many interest organizations or institutions (e.g. hospitals) are members of your organization? |
| How many volunteers work for your organization? |
| Approximately how many paid (full-time/part-time) staff work for your organization? |
| Is your organization a member of a European, international or national umbrella association?   - International (No/Yes, Please name) - European (No/Yes, Please name) - National (No/Yes, Please name) |
| In your opinion, is the number of interest organizations attempting to influence decision-making and legislation in your area increasing, decreasing or stable over the past 10–15 years?   - Increasing - Decreasing - Stable |
| Approximately how often does your organization consult with political parties?   - Never - Annually - Biannually - Monthly - Weekly |
| If possible, please specify which political parties. |
| Approximately how often does your organization consult with interest groups representing opposing interests in your area of activity?   - Never - Annually - Biannually - Monthly - Weekly |
| If possible, please specify which ones. |
| Approximately how often does your organization consult with regulatory authorities in your field of activity?   - Never - Annually - Biannually - Monthly - Weekly |
| In the last five years, approximately how many times did the government consult interest groups in your field of activity?   - Never - Annually - Biannually - Monthly - Weekly |
| How many times did your organization participate in these consultations? |
| Approximately how many times did the previous government consult interest groups in your field of activity?   - Never - Annually - Biannually - Monthly - Weekly |
| How many times did your organization participate in these consultations? |
| How difficult is it for you to access the following institutions/organizations relevant to your field of activities?   - Regulatory authorities   - Extremely difficult   - Difficult   - Sometimes possible   - Easy   - Extremely easy - Governing parties   - Extremely difficult   - Difficult   - Sometimes possible   - Easy   - Extremely easy - Opposition parties   - Extremely difficult   - Difficult   - Sometimes possible   - Easy   - Extremely easy |
| How would you describe your level of participation in parliamentary hearings/parliamentary committees?   - No participation - Low participation - Occasional participation - High participation - Very high participation |
| How would you rate the level of policy coordination/political exchange between the state and your interest group?   - Very weak - Weak - Moderate - Strong - Very strong |
| Does your organization collaborate with other like-minded organizations in the following areas?   - Fundraising   - Never   - Occasionally   - Frequently - Representation on advisory bodies   - Never   - Occasionally   - Frequently - Joint statements   - Never   - Occasionally   - Frequently - Coordinating joint political strategies   - Never   - Occasionally   - Frequently |
| Do you think that opportunities for participation in the policy process are equally distributed among interest organizations?   - Very much to the favour of other organizations - Somewhat to the favour of other organizations - Equally distributed - Somewhat to the favour of our organization - Very much to the favour of our organization |
| To what extent do you think resource-rich interest groups are overrepresented in the policy process?   - Very little - Somewhat - Very much |
| Do you experience intensive competition from organizations active in your field that represent opposing interests or values?   - Never - Usually not - Sometimes - Often - Always |
| Interest organizations often supply specialized expertise to policy-makers and political institutions. Thinking about the information or expertise you supply how would you rate the importance of the following types of information for your influence on policy?   - Technical or scientific information   - Unimportant   - Somewhat important   - Very important - Economic information   - Unimportant   - Somewhat important   - Very important - Legal information   - Unimportant   - Somewhat important   - Very important - Impact assessments   - Unimportant   - Somewhat important   - Very important |
| How has the size of your organization’s membership changed in the past 10–15 years (or since its founding, if founded more recently)?   - Decreased very much - Decreased - Stable - Increased - Increased very much |
| Does your organization have any strong ties with like-minded organizations in other EU countries? Yes/No (please specify the organizations in the comment box) |
| In recent years, have you increasingly networked with like-minded organizations abroad when trying to influence national legislation?   - Yes, very much - Yes, somewhat - No |
| What kind of support does your organization receive from related organizations abroad? (Multiple choice with comments.)   - Professional help (expertise) - Financial and material support - Training (education) of stakeholders - Preparation of joint statements and declarations about the general issues - International exchange of personnel - Other – please specify |
| What are the most important consequences of this relationship? (Multiple choice with comments.)   - Greater strength of your organization in placing issues on the domestic policy agenda - Gaining permanent consultative or partnership status in relation to government actors - Stronger inclusion of your organization in other key stages of policy-making - Transfer of knowledge, expertise and experience - Financial support - Other – please specify |
| To what extent does your organization focus on the following activities as opposed to 10–15 years ago (or since its founding, if founded more recently)?   - Organizational development   - Much less   - Less   - The same   - More   - Much more - Human resource development   - Much less   - Less   - The same   - More   - Much more - Training of lobbyists   - Much less   - Less   - The same   - More   - Much more - Fundraising   - Much less   - Less   - The same   - More   - Much more - Evaluation of efficiency and effectiveness   - Much less   - Less   - The same   - More   - Much more - Strategic planning   - Much less   - Less   - The same   - More   - Much more |
| In your view, how important are different levels of representation for your activities compared to 10–15 years ago (or since its founding, if founded more recently)?   - Local/regional   - Much less   - Less   - The same   - More   - Much more - National   - Much less   - Less   - The same   - More   - Much more - European/EU   - Much less   - Less   - The same   - More   - Much more - International   - Much less   - Less   - The same   - More   - Much more |
| To what extent do you assess the ability of your organization to assert its interests as opposed to 10–15 years ago (or since its founding, if founded more recently)?   - Much less than before - Less than before - The same - Greater now - Much greater now |
| Finally, we are wondering about the financial health of your organization. How would you assess your financial planning horizon?   - Financially stable for less than one year - Financially stable for one to two years - Financially stable for three to five years - Financially stable for about five years - Financially stable for more than five years |
| Please indicate the approximate proportion of these sources of funding in your entire budget. (Multiple choice with comments.)   - Member fees - Donations - Subsidies/grants from national governments - Commercial and marketing activities - European Union funds/projects - Other foundations/institutions/organizations |
