# Supplementary material for: Organized interests in post-communist policy-making: a new dataset for comparative research
Source: Interest Groups Advocacy. 2022 Nov 15;12(1):73–101. doi: 10.1057/s41309-022-00172-1 (PMC9665044; doi:10.1057/s41309-022-00172-1)
Supplement: Supplementary file 10 — (PDF 1078 KB) [file 41309_2022_172_MOESM10_ESM.pdf]

OrgIntCEE Political Ecology and Survey Datasets

Codebook

Michael Dobbins
Rafael Labanino
Rafał Riedel
Szczepan Czarnecki
Brigitte Horváth
Emilia Szyszkowska

Cite as: Dobbins, Michael, Labanino, Rafael, Riedel, Rafał, Czarnecki, Szczepan, Horváth, Brigitte, and Szyszkowska, Emilia (2022): "Organized Interests in Post-Communist Policy-Making: New Datasets for Comparative Research". *Interest Groups & Advocacy*

The OrgIntCEE Political Ecology Dataset contains the formation and dissolution dates between 1989 and 2019 – as well as additional information (the represented issue/subfield and constituency) – of Czech, Hungarian, Polish, and Slovenian energy policy, healthcare, and higher education civil society organizations active at the national level at any time period within the timeframe. The OrgIntCEE Survey Dataset is a survey of the active groups in these populations conducted in 2019-2020. For the documentation on the conceptual design of the research project, the sampling procedure and rules for the population ecology dataset, the response rates and the fieldwork period for the survey, please refer to the above article (open access). The English translation of the questionnaire is available as an online appendix to the article. The Czech, Hungarian, Polish and Slovenian versions of the survey can be provided by Michael Dobbins on request.

The two datasets were compiled as part of the project "The Missing Link: Examining organized interests in post-communist policy-making" at the University of Konstanz, Germany and the University of Opole, Poland. The project received generous funding from the Deutsche Forschungsgemeinschaft and the Narodowe Centrum Nauki through the Beethoven II research funding mechanism.

Content

OrgIntCEE Political Ecology Dataset variables **2**

OrgIntCEE Survey Dataset variables **3**

- ***Metadata*** *3*
- ***General variables (demographics and density)*** *3*
- ***Interest intermediation*** *8*
- ***Access and Influence*** *12*
- ***Cooperation and networking (domestic, EU, international)*** *18*
- ***Organizational development (professionalization and financial resources)*** *24*

OrgIntCEE Political Ecology Dataset variables

Variable	Variable label	Values	Value labels
name_native	Organization name in native language		
name_eng	Organization name in English		
year	Formation year		
dissolution	Year of dissolution		
type	Type of organization	1	Business
		2	Employee
		3	institutional (he, hc)
		4	consumers (en) / patients (hc) / students (he)
		5	medical doctors (hc)/employers/professionals (hc, he)/scientific promotion (en)
web	Website		
cntry	Country	1	Czech Republic / Czechia
		2	Hungary
		3	Poland
		4	Slovenia
sbfld	Subfield	1	fossil energy
		2	general energy policy
		3	nuclear energy
		4	environmental protection
		5	renewable energy
		6	healthcare general
		7	healthcare industry
		8	pharmaceutical
		9	healthcare prevention
		10	healthcare specialized
		11	higher education general
		12	higher education specialized
plfld	Policy field	1	Energy policy
		2	Healthcare
		3	Higher education
id	Unique identifier	simply a number from 1... ->	

OrgIntCEE Survey Dataset variables

Metadata

variable	question/code	code key/comment
**no**	e.g. 1, 2, 3…	simply a number from 1... ->
**id**	e.g. plhc01	cntry & pfield & no
**last_page**	1, 2, 3	the page of the online survey to which the organizations came in answering the online survey

General variables (demographics and density)

variable	question/code	code key/comment
**cntry** (country)	cz	Czechia
	hu	Hungary
	pl	Poland
	sl	Slovenia
**pfield** (policy field)	en	energy policy
	hc	healthcare policy
	he	higher education policy
**year**	e.g. 1985	founding year
**subfield**	he_gen	General higher education interests (institutional, teachers', students')

he_spec	Organizations representing the interests of scientific fields, subfields, even if scientific (e.g. Geography Society)
hc_gen	General healthcare policy interest representation (institutional, doctors, nurses, patients etc.)
hc_spec	Organizations representing the interests of a specific medical profession or patients' group, even if scientific (e.g. Society of Cardiovascular Surgery, Association of Psychiatry). Note: patients' groups such as Cancer League, representing all cancer patients should be coded as general, code here only specific groups
hc_pharm	Pharmaceutical organizations (both as medical professions, labour unions and business groups)
hc_ind	Other healthcare industry than pharmaceutical (e.g. medical equipment producers)
hc_prev	Health prevention organizations (e.g. drugs)
hc_alt	Homeopathy, Naturopathy, etc.
en_gen	General energy interest organizations
en_fossil	Fossil energy
en_re	Renewable energy
en_nu	Nuclear energy
en_prot	Green civil organizations (nature protection)

**type1 & type2**	1	Business
	2	employees
	3	employers (en) /institutional (he, hc)
	4	consumers (en) / patients (hc) / students (he)
	5	medical doctors (hc) /employers (en) /professionals (hc, en)
**conc**	1	concentrated interests: manually coded; groups representing medical doctors and the medical/pharmaceutical industry, private hospitals and healthcare technology; energy producers and suppliers (fossil, nuclear, renewables), interest representing the academic profession and lobbyists for the university system
	0	diffuse interests: manually coded; patients', healthcare awareness and employees' organizations and student organizations, consumers' groups in energy policy, groups promoting clean and sustainable energy as well as energy efficiency and energy employees' organizations
**con**	text	justification for why the organization was coded as concentrated/diffuse
**notes**	text	Notes
**memind**	How many individuals are members of your organization?<br>e.g., 0, 1, etc.	

**memch**	How has the size of your organization's membership changed in the past 10-15 years (or since its founding, if founded more recently)?	
	1	decreased very much
	2	decreased
	3	stable
	4	increased
	5	increased very much
**memch_com**	text	comment of the surveyed subject
**memfirm**	How many firms are members of your organization?	
	e.g. 0, 1, etc.	
**memint**	How many institutions are members of your organization?<br>e.g. 0, 1, etc.	
**mem_com**	text	comment of surveyed subjects on membership
**umb**	Are you an umbrella organization?	
	0	No
	1	Yes
**umb_com**	text	comment of surveyed subject
**volu**	How many volunteers work for your organization?	
	e.g. 0, 1, etc.	

**volu_com**	Text	comment of surveyed subject
**umbeu**	Is your organization a member of a European, international or national umbrella association? [Yes, European (please name)]	
	0	no
	1	yes
**umbeu_name**	Text	name of European umbrella organization
**umbint**	Is your organization a member of a European, international or national umbrella association? [Yes, international (please name)]	
	0	no
	1	yes
**umbint_name**	Text	name of international umbrella organization
**umbnat**	Is your organization a member of a European, international or national umbrella association? [Yes, national (please name)]	
	0	no
	1	yes
**umbnat_name**	text	name of national umbrella organization
**umb-eu-int-nat_com**	text	comment of surveyed subject

**staff**	number	Approximately how many paid (full-time/part-time) staff work for your organization?
**staff_com**	text	comment of surveyed subject
**intorgnum**	In your opinion, is the number of interest organizations attempting to influence decision-making and legislation in your area increasing, decreasing or stable over the past 10-15 years?	
	1	strongly decreasing
	2	decreasing
	3	stable
	4	increasing
	5	strongly increasing
**intorgnum_com**	text	comment of surveyed subject

Interest intermediation

variable	question/code	code key/comment
**consparty**	Approximately how often does your organization consult with political parties?	
	1	never
	2	annually
	3	bi-annually
	4	monthly
	5	weekly

**consparty_name**	text	If possible, please specify which political parties.
**consrival**	Approximately how often does your organization consult with interest groups representing opposing interests in your area of activity?	
	1	never
	2	annually
	3	bi-annually
	4	monthly
	5	weekly
**consrival_com**	Text	comment of surveyed subject
**consrival_name**	Text	If possible, please specify which ones.
**consreg**	Approximately how often does your organization consult with regulatory authorities in your field of activity?	
	1	never
	2	annually
	3	bi-annually
	4	monthly
	5	weekly
**consreg_com**	text	comment of surveyed subject

**govcons**	In the last five years, approximately how many times did the government consult interest groups in your field of activity?	
	1	never
	2	annually
	3	bi-annually
	4	monthly
	5	weekly
**govcons_com**	text	comment of surveyed subject
**part_govcons**	How many times did your organization participate in these consultations?	comment of surveyed subject
**part_govcons_orig-data**	text	comment of surveyed subject
**prev_govcons**	Approximately how many times did the previous government consult interest groups in your field of activity?	
	1	never
	2	annually
	3	bi-annually
	4	monthly
	5	weekly
**prev_govcons_com**	text	comment of surveyed subject
**prevpart_govcons**	How many times did your organization participate in these consultations?	comment of surveyed subject

**cz_ods_cons** (only Czech survey!)	If you can remember: approximately how many times did the previous ODS government consult interest groups in your field of activity?	comment of surveyed subject
	1	never
	2	annually
	3	bi-annually
	4	monthly
	5	weekly
**cz_ods_cons_com** (only Czech survey!)	text	comment of surveyed subject
**opport**	Do you think that opportunities for participation in the policy process are equally distributed among interest organizations?	
	1	very much to the favour of other organizations
	2	somewhat to the favour of other organizations
	3	equally distributed
	4	somewhat to the favour of our organization
	5	very much to the favour of our organization
**opport_com**	text	comment of the surveyed subject
**rich**	To what extent do you think resource-rich interest groups are overrepresented in the policy process?	
	1	very little
	2	somewhat

	3	very much
**rich_com**	text	comment of the surveyed subject
**compet**	Do you experience intensive competition from organizations active in your field that represent opposing interests or values?	
	1	never
	2	usually not
	3	sometimes
	4	often
	5	always
**compet_com**	text	comment of the surveyed subject

Access and Influence

variable	question/code	code key/comment
**regacces**	How difficult is it for your organization to access regulatory authorities?	
	1	extremely difficult
	2	difficult
	3	sometimes possible
	4	easy
	5	extremely easy

**govpaccess**	How difficult is it for your organization to access governing parties?	
	1	extremely difficult
	2	difficult
	3	sometimes possible
	4	easy
	5	extremely easy
**oppaccess**	How difficult is it for your organization to access opposition parties?	
	1	extremely difficult
	2	difficult
	3	sometimes possible
	4	easy
	5	extremely easy
**parlaccess**	How would you describe your level of participation in parliamentary hearings/parliamentary committees?	
	1	no participation
	2	low participation
	3	occasional participation
	4	high participation
	5	very high participation
**parlaccess_com**	text	comment of surveyed subject

policor	How would you rate the level of policy coordination/political exchange between the state and your interest group?	
	1	very weak
	2	weak
	3	moderate
	4	strong
	5	very strong
policor_com	text	comment of surveyed subject
fund_coop	Cooperation with other interest organizations in fundraising?	
	1	never
	2	occasionally
	3	frequently
adv_coop	Cooperation with other interest organizations in representation on advisory boards?	
	1	never
	2	occasionally
	3	frequently
statm_coop	Cooperation with other interest organizations in joint statements?	
	1	never
	2	occasionally
	3	frequently

**strat_coop**	Cooperation with other interest organizations in joint political strategies?	
	1	never
	2	occasionally
	3	frequently
**sci_expert**	Importance of provided technical or scientific expertise/information on your influence on policy?	
	1	unimportant
	2	somewhat important
	3	very important
**econ_expert**	Importance of provided economic expertise/information on your influence on policy?	
	1	unimportant
	2	somewhat important
	3	very important
**legal_expert**	Importance of provided legal expertise/information on your influence on policy?	
	1	unimportant
	2	somewhat important
	3	very important
**impact_expert**	Importance of provided impact assessment expertise/information on your influence on policy?	
	1	unimportant
	2	somewhat important
	3	very important

**reg_repr**	Local/regional - In your view, how important are different levels of representation for your activities compared to 10-15 years ago (or since its founding, if founded more recently)?	
	1	much less
	2	less
	3	the same
	4	more
	5	much more
**nat_repr**	National - In your view, how important are different levels of representation for your activities compared to 10-15 years ago (or since its founding, if founded more recently)?	
	1	much less
	2	less
	3	the same
	4	more
	5	much more
**eu_repr**	EU - In your view, how important are different levels of representation for your activities compared to 10-15 years ago (or since its founding, if founded more recently)?	
	1	much less
	2	less
	3	the same

	4	more
	5	much more
**int_repr**	International - In your view, how important are different levels of representation for your activities compared to 10-15 years ago (or since its founding, if founded more recently)?	
	1	much less
	2	Less
	3	the same
	4	more
	5	much more
**infl**	To what extent do you assess the ability of your organization to assert its interests as opposed to 10-15 years ago (or since its founding, if founded more recently)?	
	1	much less than before
	2	less than before
	3	the same
	4	greater now
	5	much greater now
**infl_com**	text	comment of the surveyed subject

Cooperation and networking (domestic, EU, international)

Variable	question/code	code key/comment
conteu	Does your organization have any strong ties with like-minded organizations in other EU countries?	
	0	No
	1	Yes
conteu_yes_com	text	comment of the surveyed subject
conteu_no	Does your organization have any strong ties with like-minded organizations in other EU countries? [no]	
	0	no to the no answer option
	1	yes to the no answer option
conteu_no_com	text	comment of the surveyed subject
conteu_na	Does your organization have any strong ties with like-minded organizations in other EU countries? [n/a]	
	0	no to n/a answer option
	1	yes to n/a answer option
conteu_com	text	comment of the surveyed subject

netw	In recent years, have you increasingly networked with like-minded organizations abroad when trying to influence national legislation?	
	1	No
	2	yes, somewhat
	3	yes, very much
netw_com	text	comment of the surveyed subject
supp_1 --> supp_6	What kind of support does your organization receive from related organizations abroad?	
	1	professional help (expertise)
	2	financial and material support
	3	training (education) of stakeholders
	4	preparation of joint statements and declarations about the general issues
	5	international exchange of personnel
	6	other - please specify
supp1	What kind of support does your organization receive from related organizations abroad? [Professional help (expertise)]	
	no	0
	yes	1
supp1_com	text	comment of the surveyed subject

supp2	What kind of support does your organization receive from related organizations abroad? [Financial and material support]	
	no	0
	yes	1
supp2_com	text	comment of the surveyed subject
supp3	What kind of support does your organization receive from related organizations abroad? [Training (education) of stakeholders]	
	no	0
	yes	1
supp3_com	text	comment of the surveyed subject
supp4	What kind of support does your organization receive from related organizations abroad? [Preparation of joint statements and declarations about the general issues]	
	no	0
	yes	1
supp4_com	text	comment of the surveyed subject
supp5	What kind of support does your organization receive from related organizations abroad? [International exchange of personnel]	
	no	0

	yes	1
supp5_com	text	comment of the surveyed subject
supp6	What kind of support does your organization receive from related organizations abroad? [Other - please specify]	
	no	0
	Yes	1
supp6_com	text	comment of the surveyed subject
supp_conseq_old	What are the most important consequences of this relationship?	
	1	greater strength of your organization in placing issues on domestic policy agenda
	2	gaining permanent consultative or partnership status in relation to governmental actors
	3	stronger inclusion of your organization in other key stages of policy making
	4	transfer of knowledge, expertise and experience
	5	financial support
	6	other - please specify
supp_conseq_old_com	text	comment of the surveyed subject

**supp_conseq_1 --> supp_conseq_6**	What are the most important consequences of this relationship?	
	1	greater strength of your organization in placing issues on domestic policy agenda
	2	gaining permanent consultative or partnership status in relation to governmental actors
	3	stronger inclusion of your organization in other key stages of policy making
	4	transfer of knowledge, expertise and experience
	5	financial support
	6	other - please specify
**conseq_angenda**	What are the most important consequences of this relationship? [Greater strength of your organization in placing issues on the domestic policy agenda]	
	0	no
	1	Yes
**conseq_angenda_com**	Text	comment of the surveyed subject
**conseq_partner**	What are the most important consequences of this relationship? [Gaining permanent consultative or partnership status in relation to government actors]	
	0	no
	1	yes
**conseq_partner_com**	text	comment of the surveyed subject

**conseq_inclusion**	What are the most important consequences of this relationship? [Stronger inclusion of your organization in other key stages of policy-making]	
	0	no
	1	yes
**conseq_inclusion_com**	text	comment of the surveyed subject
**conseq_knowledge**	What are the most important consequences of this relationship? [Transfer of knowledge, expertise and experience]	
	0	no
	1	yes
**conseq_knowledge_com**	text	comment of the surveyed subject
**conseq_financ**	What are the most important consequences of this relationship? [Financial support]	
	0	no
	1	yes
**conseq_finance_com**	text	comment of the surveyed subject
**conseq_other**	What are the most important consequences of this relationship? [Other. Please specify in comment box]	
	0	no
	1	yes

**conseq_other_com**	Text	comment of the surveyed subject

Organizational development (professionalization and financial resources)

Variable	question/code	code key/comment
org_focus	Organizational development - To what extent does your organization focus on the following activities as opposed to 10-15 years ago (or since its founding, if founded more recently)?	
	1	much less
	2	less
	3	the same
	4	more
	5	much more
hr_focus	Human resource development - To what extent does your organization focus on the following activities as opposed to 10-15 years ago (or since its founding, if founded more recently)?	
	1	much less
	2	less
	3	the same
	4	more
	5	much more

lobby_focus	Training of lobbyists - To what extent does your organization focus on the following activities as opposed to 10-15 years ago (or since its founding, if founded more recently)?	
	1	much less
	2	less
	3	the same
	4	more
	5	much more
fr_focus	Fundraising - To what extent does your organization focus on the following activities as opposed to 10-15 years ago (or since its founding, if founded more recently)?	
	1	much less
	2	less
	3	the same
	4	more
	5	much more
eval_focus	Evaluation of efficiency and effectiveness - To what extent does your organization focus on the following activities as opposed to 10-15 years ago (or since its founding, if founded more recently)?	
	1	much less
	2	less
	3	the same
	4	more
	5	much more

**plan_focus**	Strategic planning - To what extent does your organization focus on the following activities as opposed to 10-15 years ago (or since its founding, if founded more recently)?	
	1	much less
	2	less
	3	the same
	4	more
	5	much more
**prof_other**	Text	comment of the surveyed subject
**finance**	Finally, we are wondering about the financial health of your organization. How would you assess your financial planning horizon?	
	1	financially stable for less than 1 year
	2	financially stable for 1-2 years
	3	financially stable for 3-5 years
	4	financially stable for about 5 years
	5	financially stable for more than 5 years
**finance_com**	text	comment of the surveyed subject
**(funds)**	Please indicate the approximate proportion of these sources of funding in your entire budget.	
**memb_fees**	indicated in %	member fees
**donat**	indicated in %	donations
**subsid**	indicated in %	subsidies/grants from national governments

**comerc_market**	indicated in %	commercial and marketing activities
**EU_funds**	indicated in %	European Union funds/projects
**funds_other**	indicated in %	other foundations/institutions/organizations
**funds_com**	text	comment of the surveyed subject
**encomp**	0	no
	1	yes
